# Provisional Artificial Limbs

**Published:** 1918-10-19

**Authors:** 


### PROVISIONAL ARTIFICIAL LIMBS.

SEVERAL Army Council Instructions and many references in the press have appeared recently on the subject of artificial limbs. It soon became obvious that as a result of the terrible effects of modern weapons of war, and the tremendous multitudes placed in battle array, there would be an unprecedented number of men disabled by the loss of a limb or limbs. This particular class of injury excites more public emotion than any other, even than blinding, for it is conspicuous and the sufferers are about amongst their fellows in intimate association. The greatest efforts were therefore made to fit every case as soon as possible with a substitute for the member lost, and substitutes of the most expensive and elaborate kind. There seems to have been the mistake of too great hurry to supply the permanent artificial limb. Sufficient consideration was not given to the changes that take place in any stump after healing, and to the great length of time these changes involve. The result, as every Local Pensions Committee has discovered, is that a great number of men are going about possessed of artificial limbs, of the most perfect description known to limb-makers, that do not fit them, and which they either wear with discomfort and much padding or do not wear at all, preferring crutches, and great expense is incurred for alterations. Moreover, the calls on the limb-makers are so great and the trade so limited in numbers that it is difficult to keep pace with the demand.

The Ministry of Pensions, the Army Council, and the Red Cross are now all in association trying to make good the deficiency, and to obviate the need of alterations, by supplying provisional limbs of several sorts. One sort is the plaster pylons so fully described in the July issues of THE HOSPITAL. Another is of fibre. Of the fibre legs there are many kinds, and many experiments are still being made. So far the work has been done without pay by many voluntary workers at War Supply Depôts, but the demand is so great that it is doubtful if enough persons with the requisite skill, who are able to give their time and labour without pay, can be found.

It is very desirable to get all men who have lost a leg on to some form of artificial leg at the earliest possible moment, because so soon as he has a leg a man can begin to work, and idleness is not good for body or mind. Whilst a man relies on crutches he is apt to make them an excuse for loafing, and it is obvious that he cannot use his hands for work whilst he is using them for progression. Local Pensions Committees, medical men, all interested in the welfare of the wounded should use every endeavour to encourage every man who has lost a leg to get on to an artificial limb and off his crutches as soon as possible. It takes a little time to learn to use with confidence any sort of artificial limb, and much disappointment comes to men when they find that the limb which they have is ceasing to fit. The psychology of the maimed is peculiar, and the tendency often is to discard the limb and take again to crutches and a loafing existence. This mentality requires careful and very tactful combating. Medical men by explaining the certainty of changes in the stump and the certainty of eventual stability of the shape of the stump can do much to reconcile the disappointed and to inspire hope. Delay in a final provision of an artificial leg and the preliminary supply of a temporary limb may be a wise measure, for a man does not feel disappointment when what he knows is only a temporary apparatus ceases to fit him. He does feel disappointment when that limb which he believed would finally enable him to take up his old way of life again, or a new way, fails to realise his expectations.

It will be interesting to note in a few years' time whether many men are still using the ingenious and cleverly made permanent limbs now supplied, or have given them up in favour of a simple peg-leg. Many medical men know how active old amputation cases are with the simple, old-fashioned peg, and have had experience of men discarding a more shapely but more cumbrous limb in favour of a wooden leg. When a man has to wear an artificial leg for hours on end walking or working he is likely to prefer lightness to appearance.

